# An international survey on aminoglycoside practices in critically ill patients: the AMINO III study

**DOI:** 10.1186/s13613-021-00834-4

**Published:** 2021-03-19

**Authors:** Claire Roger, Benjamin Louart, Loubna Elotmani, Greg Barton, Leslie Escobar, Despoina Koulenti, Jeffrey Lipman, Marc Leone, Laurent Muller, Caroline Boutin, Julien Amour, Iouri Banakh, Joel Cousson, Jeremy Bourenne, Jean-Michel Constantin, Jacques Albanese, Jason A. Roberts, Jean-Yves Lefrant, The Azurea Network, Bruno Darchy, Lasocki Sigismond, Karim Debbat, Sébastien Moschietto, Gilles Capellier, Matthieu Biais, Guillaume Brunin, Olivier Huet, Marie-Hélène Hausermann, Bernard Just, Jean-Michel Constantin, Jean-François Payen, Géraldine Dessertaine, Pierre Lavagne, Arnaud Winer, Olivier Lesieur, Mickaël Landais, Arnaud Friggeri, Jules Piclet, Bruno Pastène, Jacques Albanèse, Jérémy Bourenne, Frédéric Bellec, Samir Jaber, Pierre Egreteau, Marie-Reine Losser, Karim Asehnoune, Karim Lakhal, Carole Ichai, Bernard Goubaux, Laurent Muller, Caroline Boutin, Matthieu Legrand, Julien Amour, Philippe Montravers, David Marrache, Walter Picard, Claire Dahyot-Fizelier, Joël Cousson, Philippe Seguin, Pascal Beuret, Arnaud Delahaye, Benoit Veber, Jérôme Morel, Ali Mofredj, Nicolas Barbarot, Bernard Georges, Henri Faure, Pierre Saint Léger, Iouri Banakh, Jeffrey Lipman, Paul Williams, Manimozhi Vellaichamy, Maria Downey, Ruth Rosales, Roberto Amador, Daniel Muñoz, Marcial Cariqueo, Metaxia Papanikolaou, Anna Spring, Evdoxia Tsigou, Vasilios Koulouras, Polychronis Tasioudis, Greg Barton, Emma Graham Clarke, Richard Bourne, Mark Borthwick, Mark Tomlin, Emma Boxall, Catherine Mc Kenzie, Ruth Roadley-Battin, Timothy Felton

**Affiliations:** 1 Department of Intensive Care Medicine, Division of Anesthesiology, Intensive Care, Pain and Emergency Medicine, Nîmes University Hospital, Place du Professeur Robert Debré, 30 029 Nîmes cedex 9, France; 2 Equipe D, Caractéristiques Féminines Des Interfaces Vasculaires (IMAGINE), Faculté de Médecine, Univ Montpellier, 2992 Montpellier, France; 3 St Helens and Knowsley Hospitals NHS Trust, Liverpool, UK; 4 Faculty of Medicine, Universidad de Chile, Santiago, Chile; 5 The University of Queensland Centre for Clinical Research, The University of Queensland, Brisbane, Australia; 6 Department of Intensive Care Medicine, Royal Brisbane and Women’s Hospital, Brisbane, Australia; 7 Department of Anesthesiology and Intensive Care Medicine, University Hospital of Marseille, Marseille, France; 8 Institute of Perfusion, Critical Care Medicine and Anesthesiology in Cardiac Surgery (IPRA), Hôpital Privé Jacques Cartier, Massy, France; 9 Frankston Hospital, Frankston, VIC Australia; 10 Department of Anesthesiology and Intensive Care Medicine, University Hospital of Reims, Reims, France; 11 Department of Emergency and Intensive Care Medicine, University Hospital of Marseille, Hôpital de La Timone, Marseille, France; 12 Department of Anesthesiology and Intensive Care Medicine, University Hospital of Clermont-Ferrand, Clermont-Ferrand, France; 13 Department of Anesthesiology and Intensive Care Medicine, University Hospital of Marseille, Hôpital de La Conception, Marseille, France; 14 Pharmacy Department, Royal Brisbane and Women’s Hospital, Brisbane, Australia; 15 Second Critical Care Department, Attikon University Hospital, Athens, Greece

**Keywords:** Antibiotics, Aminoglycoside, ICU, Therapeutic drug monitoring, PK/PD

## Abstract

**Background:**

While aminoglycosides (AG) have been used for decades, debate remains on their optimal dosing strategy. We investigated the international practices of AG usage specifically regarding dosing and therapeutic drug monitoring (TDM) in critically ill patients. We conducted a prospective, multicentre, observational, cohort study in 59 intensive-care units (ICUs) in 5 countries enrolling all ICU patients receiving AG therapy for septic shock.

**Results:**

We enrolled 931 septic ICU patients [mean ± standard deviation, age 63 ± 15 years, female 364 (39%), median (IQR) SAPS II 51 (38–65)] receiving AG as part of empirical (761, 84%) or directed (147, 16%) therapy. The AG used was amikacin in 614 (66%), gentamicin in 303 (33%), and tobramycin in 14 (1%) patients. The median (IQR) duration of therapy was 2 (1–3) days, the number of doses was 2 (1–2), the median dose was 25 ± 6, 6 ± 2, and 6 ± 2 mg/kg for amikacin, gentamicin, and tobramycin respectively, and the median dosing interval was 26 (23.5–43.5) h. TDM of *C*_max_ and *C*_min_ was performed in 437 (47%) and 501 (57%) patients, respectively, after the first dose with 295 (68%) patients achieving a *C*_max_/MIC > 8 and 353 (71%) having concentrations above *C*_min_ recommended thresholds. The ICU mortality rate was 27% with multivariable analysis showing no correlation between AG dosing or pharmacokinetic/pharmacodynamic target attainment and clinical outcomes.

**Conclusion:**

Short courses of high AG doses are mainly used in ICU patients with septic shock, although wide variability in AG usage is reported. We could show no correlation between PK/PD target attainment and clinical outcome. Efforts to optimize the first AG dose remain necessary.

*Trial registration* Clinical Trials, NCT02850029, registered on 29th July 2016, retrospectively registered, https://www.clinicaltrials.gov

**Supplementary Information:**

The online version contains supplementary material available at 10.1186/s13613-021-00834-4.

## Background

Early administration of adequate doses of antibiotics is a key issue for the optimal management of sepsis in critically ill patients [1, 2]. Optimized dosing is likely to reduce mortality in patients with severe sepsis and septic shock [3]. Therefore, broad-spectrum antimicrobial therapy that combines more than one antimicrobial agent can be used to ensure appropriate therapy in the initial phase of severe sepsis [4]. Even though the benefit of combination therapy remains unclear, aminoglycosides (AG) are often given as part of empirical therapy for sepsis, especially when Gram-negative bacteria are suspected [5–7]. To achieve maximal efficacy, the optimal AG dosing regimen should reach a peak concentration to minimum inhibitory concentration (peak/MIC) ratio of at least 8–10, as this pharmacokinetic/pharmacodynamics (PK/PD) target has been associated with clinical cure [8–11]. On the other hand, the optimal AG dosing regimen should expose patients to the lowest risk of toxicity [12]. Therapeutic drug monitoring (TDM), trough concentration monitoring in particular, is encouraged in critically ill patients to guide re-dosing and to limit toxicity. Many factors influence the PK and PD of antimicrobials in critically ill patients. An increased volume of distribution, the occurrence of hypo-albuminemia, and renal and/or hepatic dysfunctions are frequently observed in those patients leading to altered PK [13]. Previous studies have reported that, for up to 30–40% of critically ill patients, AG plasma concentrations were lower than the targeted peak concentrations using standard dosing regimens [14–17]. Positive fluid balance during the previous day and low body mass index (BMI) have been reported as risk factors of insufficient peak plasma concentrations [17]. The use of higher amikacin dosing regimens (≥ 25 mg/kg) has been suggested by several authors and recommended by national guidelines [12, 14, 18]. However, practices of antimicrobial therapy and TDM may widely vary based on available resources and local guidelines. Moreover, as local ecology and MICs’ distribution may also differ among countries, this could lead to different practices of AG use among countries. The main objective of the study was to provide a detailed description of agents, dosing, and monitoring practices of AG in in a large cohort of critically ill patients from different countries receiving AG for a sepsis or a septic shock.

The secondary objectives were to report renal adverse events and clinical outcomes, and to assess the correlation between PK/PD target attainment and these outcomes (clinical success and ICU mortality).

## Methods

This study is reported in accordance with the STrengthening the Reporting of OBservational studies in Epidemiology (STROBE) guidelines [19].

A prospective, international, observational cohort design was used. All participating ICUs obtained local ethics committees’ approval [ethics approval for France (Institutional Review Board of Nîmes University Hospital, France 15/04.08) [20]] and the study was registered in Clinical Trials (NCT02850029). Written informed consent was waived due to the non-interventional design of the study.

### Study population

All adult patients (≥ 18 years) requiring ICU care and receiving AG (amikacin, gentamicin, and tobramycin) therapy for a sepsis according to the Third International Consensus Definitions for Sepsis and Septic Shock (Sepsis-3) were included in the study [21].

### Settings

Overall, 85 ICUs accepted to participate to the study, and 59 ICUs enrolled patients from May 2016 to August 2017 in five different countries: Australia (5), Greece (5), Chile (4), UK (9), and France (62).

### Data collection and management

#### Participating site data

For each participating ICU, the following information were recorded: type of hospital, type of ICU, number of beds, number of physicians, number of admissions per year (on the last year), annual ICU mortality, national or local AG guidelines availability, and TDM availability.

#### Patient data

Data recorded for each patient included were: demographic data (age, gender, height, total body weight), clinical data [admission diagnosis, co-morbidities, APACHE II or SAPS II (Simplified Acute Physiology Score) at admission, SOFA (Sequential Organ Failure Assessment) score at inclusion], and biological data [serum creatinine concentration, albumin concentration, and 24-h urine output every day during AG therapy (where possible)]. Additionally, dosing and TDM data when available (dose and frequency, time of dosing and sampling, therapy duration, maximal and minimal AG plasma concentrations, and co-administered antibiotics) and toxicity data [co-administered nephrotoxic drugs, renal function assessment using the Acute Kidney Injury Network (AKIN) criteria [22]] at the end of AG therapy were collected. Finally, infection data (known or presumed pathogen, known or likely MIC) and the following outcome data: clinical success, ICU and hospital length of stays (LOS), and ICU mortality were recorded. Clinical success was defined as the resolution of initial symptoms for which AG were prescribed, relapse was defined as the occurrence of a new infectious episode with the same identified pathogen, and superinfection was defined as the occurrence of a new infectious episode with a different pathogen).

The definition used to assess PK/PD outcome was as follows. PK/PD target attainment was defined as AG concentration after the first dose ≥ 8 times the MIC where AG plasma concentrations were available. Breakpoints from the EUCAST database were considered [23]. Where infections were polymicrobial, the MIC of the least susceptible pathogen was used in the analysis. Actual values of MIC were considered where available to assess “a posteriori” *C*_max_/MIC ratio.

### Data management

Data collection was performed by trained staff at each participating center. Data were entered into a structured electronic password-protected and secured web-based case report form (eCRF). The eCRF was developed using the open-source REDCap Data Management Platform hosted at the Nîmes University Hospital [24].

Data monitoring was handled by the coordinating center (Nîmes University Hospital, France). Outstanding queries regarding the completion of the CRF were undertaken with each participating center where necessary to ensure accuracy of data.

### Statistical analysis

Descriptive statistics of the primary outcome were reported using mean, standard deviation, median, and interquartile range (25^th^–75th percentile) for continuous variables. For categorical variables, frequencies and proportions were given. Two secondary outcomes were explored separately: ICU mortality and clinical success. We used multivariate regression models involving eight covariates: admission type (medical or surgery), age, gender, body mass index (BMI, kg m^−2^), SOFA score on the day of AG initiation, creatinine plasma concentration (µmol L^−1^) on the day of AG initiation, bacteremia, and directed or empirical antibiotic treatment. A 9th variable related to AG treatment was considered. We constructed a model for each way we considered AG: the mg/kg dose using either the total body weight (TBW), the ideal body weight (IBW, thanks to the Lorentz's formula), or the adjusted body weight [ABW: given by the TBW when the BMI was below 30 kg m^−2^ and when the BMI was over 30 kg m^−2^ calculated according to the following formula: IBW + 0.4 x (TBW-IBW)], the peak concentration (mg L^−1^), the attainment of a peak concentration between 60 and 80 mg L^−1^ for amikacin, and between 20 and 30 mg L^−1^ for gentamicin. Because practices could differ between hospitals, we needed a model accounting for correlation within centers. For this purpose, we chose a GEE (generalized estimating equations) model with an exchangeable correlation structure. This method allows parameters of generalized linear model to be estimated in clustered data and returns a marginal model that fits in with our purpose (i.e., estimating average response in overall population) [25, 26]. 95% Confidence intervals (CI) for odds ratio (OR) were given in the univariate and the multivariate analysis based on the robust standard error provided by the Huber–White standard error and assuming the normal distribution of the regression coefficients [27]. To test the significance of OR, we performed Wald tests. All models were built in the whole population. A *P* value of 0.05 was considered to indicate statistical significance. All statistical analyses were performed using R software (version 3.6.0) [28].

## Results

From May 2016 to August 2017, 971 patients were enrolled from 59 ICUs worldwide (Additional file 1: Figure S1). Of the 971 patients initially included in the study, 40 were excluded because of missing data leading to 931 studied patients.

### Participating site data

Most of the ICUs were from university hospitals (38, 64%), 3 were medical ICUs, 14 were surgical, and 42 were mixed-ICUs. Description of the participating sites and their local protocols regarding AG therapy are described in Additional file 2: Table S1.

### Demographic data

Patient demographic data at inclusion are presented in Table 1. The source of sepsis was pulmonary (405, 44%), abdominal (233, 25%), urinary (126, 14%) infection, or bacteremia (158, 17.0%). The most common microorganisms for which AG were prescribed are presented in Table 2. Among patients with microbiological samples drawn (*n* = 826, 89%), 511 patients had positive cultures. MICs for all administered antibiotics were available in 135 patients with 127 (94%) receiving adequate antimicrobial therapy. In 22 (17%) patients, AG enabled to broaden spectrum activity.

**Table 1:** Patients' characteristics at baseline

Variables	*n*	Value
Age (years)	929	63 ± 15
Female	928	364 (39%)
Weight (TBW, kg)	922	77 ± 20
Height (cm)	898	169 ± 9.5
BMI (kg m^−2^)	897	27 ± 6.8
Medical/surgical admission	929	512 (55%)/417 (44.8%)
SAPS II	792	51 [38;65]
APACHE	109	22 [15;28]
SOFA	921	8 [6;11]
Creatinine level (µmol L^−1^)	930	133 ± 120
Albumin (g L^−1^)	407	27 ± 7
Protein (g L^−1^)	773	55.6 ± 12.3

For continuous variables mean ± standard deviation or median [interquartile range] are given. For categorical variables, numbers (%) are given

TBW: total body weight; BMI: body mass index; ICU: intensive-care medicine

**Table 2:** Microbiological data

Variables	*n*
Microbiological sample (n = 930)	826 (89%)
Number of pathogens identified (n = 511)	
1	317 (62%)
2	121 (24%)
3 and more	73 (14%)
Pathogens (*n* = 798)	
Gram-positive bacteria	255 (32%)
Staphylococcus/*S. aureus*	120 (15%)/84 (10.5%)
Streptococci	64 (8%)
Enterococci	67 (8.4%)
Gram-negative bacteria	453 (58%)
* Escherichia coli*	166 (21%)
* Klebsiella* spp.	70 (8.8%)
* Pseudomonas aeruginosa*	73 (9%)
* Enterobacter* spp.	41 (5%)
* Acinetobacter baumannii*	9 (1%)
* Stenotrophomonas maltophila*	5 (0.6%)
Anaerobes bacteria	24 (3%)
Fungi/Candida	33 (4%)/31 (3.9%)
Susceptibility testing (*n* = 322)	
AG susceptibility categories (S/I/R)	254 (79%)/14 (4%)/54 (17%)

Results are given as numbers and percentages

AG: aminoglycoside; MIC: minimal inhibitory concentration; R: resistant; I: intermediate; S: susceptible

### Primary outcome

**Fig. 1:**
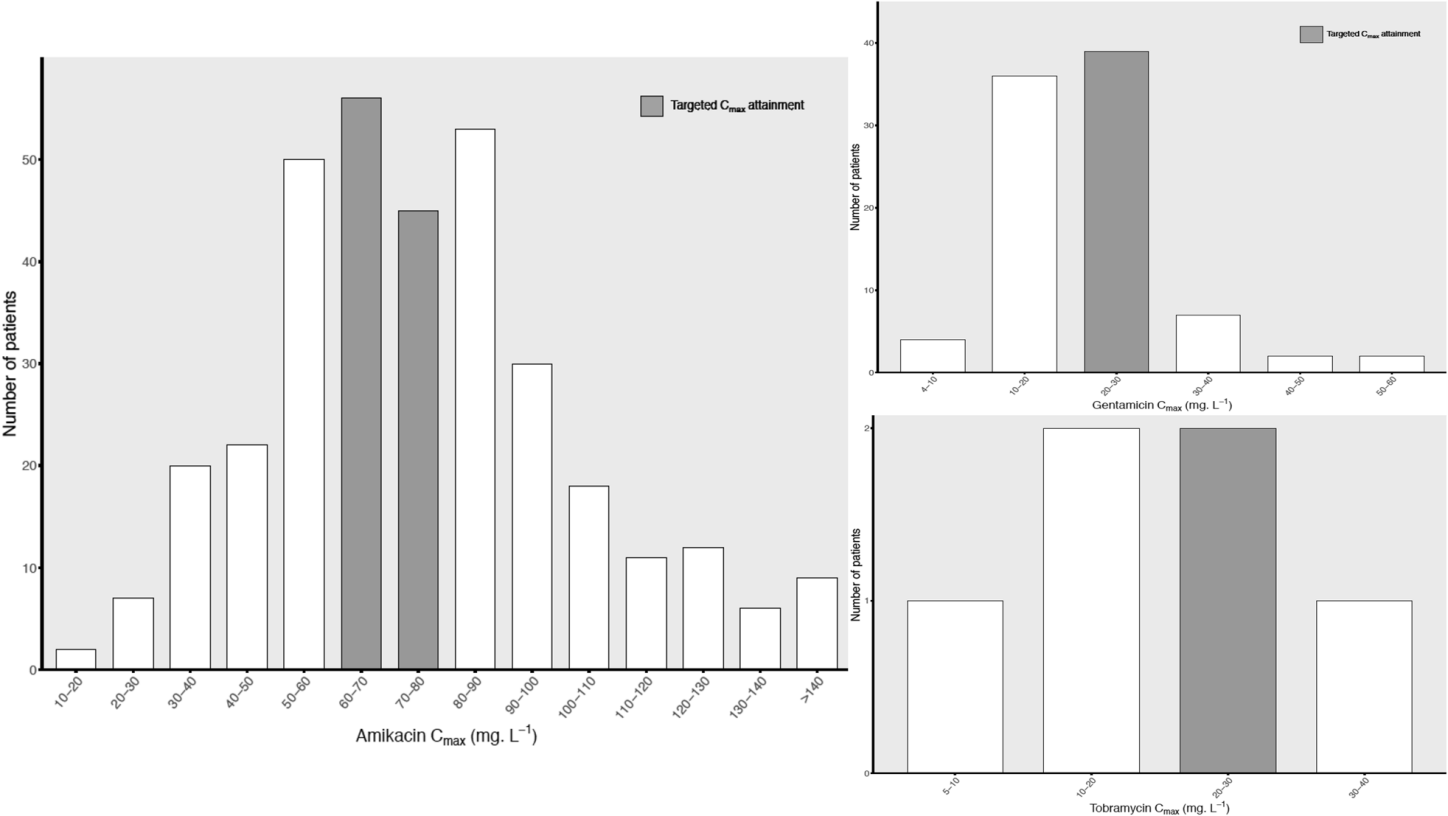
Distribution of amikacin and gentamicin *C*_max_ (maximal concentration) plasma concentrations

**Fig. 2:**
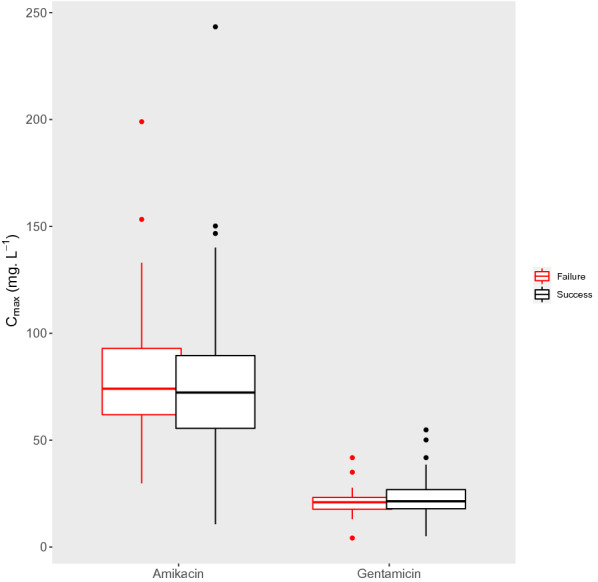
Observed amikacin and gentamicin *C*_max_ (maximal concentration) plasma concentrations according to clinical success or failure. The lower and upper hinges correspond to the first and third quartiles (the 25th and 75th percentiles). The upper whisker extends from the hinge to the largest value no further than 1.5 times the IQR from the hinge (where IQR is the interquartile range, or distance between the first and third quartiles). The lower whisker extends from the hinge to the smallest value at most 1.5 times the IQR of the hinge. Data beyond the end of the whiskers are plotted individually

Among the 931 included patients, 14 (1%) received tobramycin, 303 (33%) received gentamicin, and 614 (66%) received amikacin. Practices of AG administration are described in Table 3. AG were prescribed empirically in 761 (82%) patients and as a combination therapy in 914 (98%) patients, mostly in association with ß-lactam antibiotics (890, 96%). The median duration of therapy was 2 ((IQR 1–3) days, the median number of AG doses per course was 2 ((IQR 1–2), and the median dosing interval was 26 (IQR 23.5–43.5) h. 483 patients (52%) received a second dose. TDM of *C*_max_ and *C*_min_ concentrations was performed in 437 (47%) and 501 (57%) patients for the first dose (Figs. 1 and 2). After the first AG dose, 295 (68%) patients achieved the PK/PD target of *C*_max_/MIC > 8 considering an MIC of 8 mg L^−1^ for amikacin ((241/341, 71%), and 2 for gentamicin (53/90, 59%) and tobramycin (1/6, 17%), whereas 353 (71%) patients had concentrations above *C*_min_ recommended thresholds. For 128 patients, an MIC was available for at least one pathogen, with a median of 2 [1; 4] mg L^−1^. Among these 128 patients, 76 had both MIC and *C*_max_ available, with a median *C*_max_/MIC ratio of 25.7 [16.7; 40.3] and for 65 patients *C*_max_/MIC ratio was above 8.

**Table 3:** Aminoglycoside therapy characteristics

	All (*n* = 931)	Amikacin (*n* = 614)	Gentamicin (*n* = 303)	Tobramycin (*n* = 14)
Duration of therapy (days)	2 [1; 3]	2 [1; 3]	2 [1; 3]	2 [2; 4.75]
Number of injections	2 [1; 2]	2 [1; 2]	2 [1; 2]	2 [1; 4]
Second dose administered	483 (52%)	326 (53%)	164 (53%)	13 (93%)
First dose in mg	–	2000 [1500; 2250]	440 [320; 560]	500 [381; 555]
First dose in mg per kg of TBW	–	26 [21.9; 29.4]	5.6 [4.3; 7.5]	6 [5.1; 7.1]
First dose in mg per kg of ABW	–	267.8 [23.8; 29.4]	6.2 [4.8; 7.8]	6.1[5.4; 7.1]
Infusion duration (min)	30 [30; 30]	30 [30; 30]	30 [30; 30]	30 [30; 30]
*C*_max_ after first dose	437/931 (47%)	341/614 (56%)	90/303 (30%)	6/14 (43%)
*C*_max_ value (mg L^−1^)	–	73 [57; 90]	21 [17; 25]	19 [14; 26]
Delay end of infusion and *C*_max_ sampling (min)	30 [30; 50]	30 [30; 50]	30 [30; 44]	30 [26; 37]
Targeted *C*_max_ attainment after first dose	295/437 (68%)	241/341 (71%)	53/90 (59%)	1/6 (17%)
*C*_min_ after first dose	501/880 (57%)	347/577 (60%)	145/289 (50%)	6/14 (43%)
*C*_min_ above threshold	353/498 (71%)	230/347 (66%)	118/145 (81%)	5/6 (83%)
Time from first dose to* C*_min_ (h)	22.9 [20.4; 24]	22.6 [20.5; 24]	23.4 [21.5; 24.1]	24.4 [23.6; 26.1]
Interval after first dose (h)	26 [23; 43]	26.7 [23; 45]	24.8 [23; 34]	24.5 [23; 29]

For continuous variables, mean ± standard deviation or median [interquartile range] are given. For categorical variables, numbers (%) are given. The ABW was calculated as follow: IBW + 0.4 x (TBW-IBW). The *C*_max_ attainment is defined as a *C*_max_ above 20 mg L^−1^ for gentamicin and tobramycin or above 60 mg L^−1^ for amikacin. The *C*_min_ thresholds considered are 0.5 mg L^−1^ for gentamicin and tobramycin and 2.5 mg L^−1^ for amikacin

*C*_max_: maximal concentration; *C*_min_: minimal concentration; ABW: adjusted body weight, TBW: total body weight; IBW: ideal body weight

### Secondary outcomes

#### Clinical response

Antimicrobial therapy was considered successful in 621/843 (74%) patients. Relapse and superinfection were observed in 42/843 (5%) and 71/843 (8%) patients, respectively, whereas in 17 patients, emergence of multidrug resistant (MDR) pathogens was noted.

#### Renal function on AG therapy

Concomitantly to AG, 361 (39%) patients received at least one nephrotoxic agent among non-steroid anti-inflammatory drug, iodine, angiotensin-converting enzyme, and vancomycin. On the last day of therapy, AKIN score was $\ge 1$ in 108/481 (22%) analysable patients.

#### Patient outcome

The ICU mortality rates were 27%, respectively. The median ICU and hospital LOS were 11 (IQR 5–25) and 24 (IQR 11–43) days, respectively.

Independent factors associated with ICU mortality and clinical success are presented in Tables 4 and 5. SOFA score was independently associated with the risk of clinical failure and death. Additionally, patient age was an independent risk factor associated with ICU mortality, whereas *C*_max_ attainment was not independently associated with clinical success and ICU mortality. The different other models tested did not find a significant difference in the association between clinical outcomes and the variables of interest related to AG.

**Table 4:** Multivariate analysis of clinical success for all AG considering targeted *C*_max_ (maximal concentration) attainment

	Clinical success	
	OR (95% CI)	*P* value
Medical admission	1.08 [0.67; 1.74]	0.74
Male	0.60 [0.32; 1.12]	0.11
Age (years)	1.00 [0.99; 1.02]	0.67
BMI (kg m^−2^)	1.01 [0.97; 1.04]	0.91
**SOFA score**	*0.85 [0.79; 0.91]*	* < 0.0001*
Serum creatinine (µmoL L^−1^)	1.00 [0.99; 1.0]	0.52
Empirical treatment	0.71 [0.33; 1.53]	0.39
Bacteremia	0.87 [0.49; 1.55]	0.64
*C*_max_ attainment	1.24 [0.79; 1.94]	0.35

Italic values indicates *P* values <0.05

OR were calculated with a GEE model assuming an exchangeable correlation structure. *P* values result of a Wald test. The regression was based on 360 patients. The targeted *C*_max_ was defined as a serum level above 60 mg L^−1^ for amikacin and 20 mg L^−1^ for gentamicin and tobramycin

OR: odds ratio; CI: confidence interval, BMI: body mass index, GEE: generalized estimating equations

**Table 5:** Multivariate analysis of ICU mortality for all AG considering targeted *C*_max_ (maximal concentration) attainment

	ICU mortality	
	OR (95% CI)	*P* value
Medical admission	1.28 [0.75; 2.17]	0.36
Male	1.08 [0.73; 1.60]	0.69
Age (years)	*1.03 [1.01; 1.04]*	*0.001*
BMI (kg m^−2^)	1.00 [0.97; 1.04]	0.8
**SOFA score**	*1.18 [1.10; 1.26]*	* < 0.0001*
Serum creatinine (µmoL L^−1^)	0.99 [0.99; 1.01]	0.32
Empirical treatment	0.81 [0.45; 1.48]	0.5
Bacteremia	0.62 [0.31; 1.25]	0.18
*C*_max_ attainment	0.78 [0.46; 1.31]	0.34

Italic values indicates *P* values <0.05

OR were calculated with a GEE model assuming an exchangeable correlation structure. *P* values result of a Wald test. The regression was based on 393 patients. The targeted *C*_max_ was defined as a serum level above 60 mg L^−1^ for amikacin and 20 mg L^−1^ for gentamicin and tobramycin

OR: odds ratio, CI: confidence interval, BMI: body mass index, GEE: generalized estimating equations

## Discussion

### Main findings

Short course of high doses of AG is the dosing regimen extensively used at an international level with amikacin being the most commonly AG prescribed in this large cohort of septic patients. AG TDM is current practice for most of participating ICUs, although ICU patients for whom this monitoring is recommended vary between local guidelines. Finally, in the subgroup of patients for whom TDM was performed, we could find no correlation between *C*_max_ target attainment and ICU mortality or clinical success (Fig. 2).

### Relationship to previous papers

Although AG have been prescribed for decades, administering optimal dosing regimens in critically ill patients with sepsis or septic shock remain challenging due to several pathophysiological changes affecting drug PK and due to the emergence of resistance. Additionally, nephrotoxicity has been one of the reasons limiting AG use in critically ill patients with frequent compromised renal function. Aminoglycoside dose optimization can be defined as the dose having the highest likelihood of a good outcome and the lowest likelihood of toxicity. Consequently, doses have to be given once daily and should be stopped as early as possible [29]. Extended interval of high doses of AG has been suggested by several authors to maximize efficacy while limiting the probability of nephrotoxicity [17, 18]. The findings of the present study confirm that, in critically ill patients receiving AG therapy, this dosing regimen is widely used. Still, this higher dosing regimen is considered as an “off-label” dosing regimen in most countries. Rybak et al. have previously reported low prevalence of nephrotoxicity (1.2%) when short courses (3 days) of AG were administered [30]. In the present study, more than 20% of patients had persistent AKI at the end of treatment. Similar findings have been reported by Duszynska et al. in septic patients reporting that 24% of patients developed AKI during amikacin therapy [31]. Finally, the rate of nephrotoxicity attributable to AG therapy is difficult to evaluate as several confounders may influence the persistence of AKI in septic patients. Sepsis-related AKI ranges from 20 to 50%, independently of AG use [32].

Amikacin is the AG most being prescribed in this large cohort of critically ill patients. However, clinical practices widely vary among countries and local epidemiology. Indeed, given that the vast majority of ICUs were located in France, it may explain the use of amikacin in two thirds of patients in this cohort as amikacin is commonly prescribed in this country. Even though the number of patients receiving gentamicin was smaller in our cohort, it is interesting to note that the target attainment rate is lower (59%) than for amikacin (71%). The median dose of 6 mg kg^−1^ appears insufficient to achieve PK/PD target. Indeed, an increased volume of distribution (0.41 L kg^−1^) of gentamicin has been observed in sepsis leading to gentamicin underexposure and increasing risk of therapeutic failure [33]. Higher gentamicin loading doses should be given to critically ill patients as reported previously [34]. Similarly, in a previous single centre study, we showed that a gentamicin dosing regimen of 8 mg kg^−1^ of total body weight failed to reach the PK/PD target of at least 8 times the MIC in a significant proportion of critically ill patients considering an MIC of 2 mg L^−1^.[18] Considering actual MIC, the proportion of patients experiencing underdosing could be lower as most of the identified pathogens in this study had an MIC < 2 mg L^−1^. Nevertheless, when AG are used as empirical therapy at the early phase of septic shock where bacterial inoculum is supposed to be high and antibiotic appropriateness is essential, the worst-case scenario (i.e., covering the least susceptible pathogen) should be considered to choose optimal dosing regimen to ensure therapeutic efficacy.

AG TDM has been advocated to optimize AG drug dosing in the setting of ICU patients targeting a *C*_max_/MIC ratio of at least 8 to maximize the effect of treatment [35, 36]. In a previous study, Duszynska et al. showed that among septic patients, 83% required dose and/or interval adjustment during amikacin therapy [31]. Conversely to this study and others showing good clinical outcomes correlated to this PK/PD target, we failed to demonstrate any correlation between *C*_max_ target attainment and clinical outcome in the present study (Fig. 2) [37]. One potential explanation could be the wide use of combination therapy with beta lactams to treat severe infections. The impact of non-target attainment for AG could be minimized by the administration of appropriate combined antibiotic questioning the need for adding AG in this setting. However, it is noteworthy that AG were the only antibiotics effective against the causative agent for 22 patients. Also, recent guidelines on the treatment of sepsis have recommended using combination therapy, especially in patients with septic shock [38, 39]. Consequently, it is important to optimize AG dosing as if they were the only effective agent being used. Unfortunately, the small size of this cohort of patients for whom AG enabled to broaden spectrum activity precluded to identify any correlation between target attainment and clinical outcome. Another important finding is the significant proportion (67%) of patients that still did not achieve PK/PD targets in our cohort, even though high doses of AG were mostly used. Previously, some authors aimed to identify the main determinants of the first amikacin peak in critically ill patients [40]. Based on their findings, Boidin et al. supported the use of an amikacin dose ≥ 37.8 mg kg^−1^ of lean body weight (LBW) to optimize the attainment of *C*_max_  ≥ 80 mg L^−1^ after the first dose in critically ill patients [40]. As interindividual *C*_max_ variability has been observed in several previous studies, it is unlikely that the “one dose fits all” strategy would be the optimal strategy in this population. Conversely, TDM should be considered even when short courses of AG are administered. TDM has been shown to decrease hospital stay, and the incidence of nephrotoxicity, mortality, and costs [41]. However, TDM alone may not be sufficient to determine the appropriate dosing to be given. Individualized approaches using Bayesian forecasting and TDM algorithm should be implemented in daily practice to take into account the different factors affecting AG PK in septic patients [41, 42]. These approaches also offer the advantage to estimate area under the curve (AUC) in clinical practice with limited PK sampling. Estimates of *C*_max_ can vary substantially based on the duration of the infusion and the timing of *C*_max_ sampling, whereas AUC appears more reliable and stable measure. Additionally, AUC better reflects cumulative exposure over the entire dosing period and may be less sensitive to differences in concentration sampling times. Thus, AUC-guided TDM has been advocated as a more reliable indicator of bacterial killing and clinical efficacy for AG [43].

### Study limitations

Several limitations of the AMINO III study needs to be acknowledged.

First, the distribution of the participating ICUs may not reflect the daily practice of all ICUs for each country as in some countries a small number of patients were included. Second, PK/PD outcome assessment was possible in less than half of the included patients. As a result, correlations with patient and site characteristics factors may have been underestimated. However, these findings highlight that, despite national or local recommendations, aminoglycoside TDM is not systematically performed in septic patients. Third, we only considered AG use in septic patients in the present study precluding to extrapolate these findings to other indications of AG therapy. Fourth, assessment of clinical success was not assessed by an independent committee; however, it was clearly predefined and standardized for improving data quality. Finally, ototoxicity was not assessed due to the observational design of the present study.

## Conclusion

Short courses of high AG doses are mainly used in ICU patients with septic shock, although wide variability in AG usage is reported. In this cohort, we could show no correlation between *C*_max_ target attainment and ICU mortality or clinical success. Efforts to optimize the first AG dose and to perform TDM are still needed.

## Supplementary Information

**Additional file 1: Figure S1.** Representation of AMINO III study participating sites. Dark gray: study participating countries

**Additional file 2: Table S1.**

## Data Availability

The dataset used and analyzed during the current study are available from the corresponding author on reasonable request.

## Abbreviations

AG: Aminoglycosides
TDM: Therapeutic drug monitoring
ICU: Intensive-care unit
PK/PD: Pharmacokinetics/pharmacodynamics
MIC: Minimal inhibitory concentration
BMI: Body mass index
AKIN: Acute Kidney Injury Network
CRF: Case report form
TBW: Total body weight
IBW: Ideal body weight
ABW: Adjusted body weight
*C*_max_: Maximal plasma concentration
*C*_min_: Minimal plasma concentration
MDR: Multidrug resistant
LBW: Lean body weight
AUC: Area under the curve
