# Supplementary figures and images for: An international survey on aminoglycoside practices in critically ill patients: the AMINO III study
Source: Ann Intensive Care. 2021 Mar 19;11:49. doi: 10.1186/s13613-021-00834-4 (PMC7979853; doi:10.1186/s13613-021-00834-4)

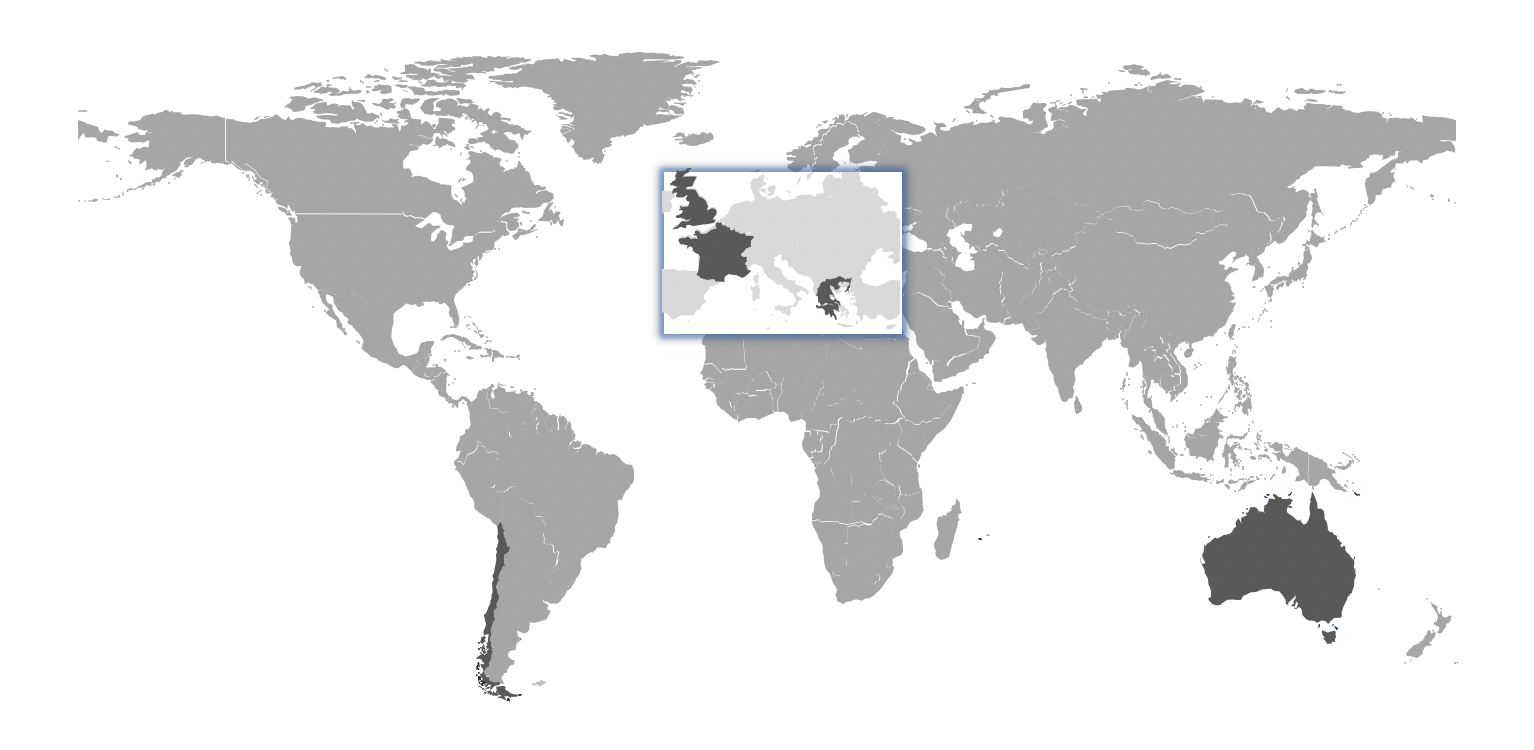

Supplement: Supplementary file 1 — Additional file 1: Figure S1. Representation of AMINO III study participating sites. Dark gray: study participating countries [file 13613_2021_834_MOESM1_ESM.png]
