# Supplementary material for: An international survey on aminoglycoside practices in critically ill patients: the AMINO III study
Source: Ann Intensive Care. 2021 Mar 19;11:49. doi: 10.1186/s13613-021-00834-4 (PMC7979853; doi:10.1186/s13613-021-00834-4)
Supplement: Supplementary file 2 — Additional file 2: Table S1. [file 13613_2021_834_MOESM2_ESM.docx]

Supplemental material S2.

|  | N (%) or median (IQR) |
| --- | --- |
| Number of ICU beds per ICU  <10  10-15  15-20  >20 | 5 (9%)  24 (42%)  16 (28%)  12 (21%) |
| Number of ICU admissions per year | 800 (580-991) |
| Number of ICU physicians per unit | 7 (5-9) |
| Number of nurse per bed ratio | 0.4 (0.4-0.42) |
| One year mortality | 20 (16-24%) |
| Therapeutic drug monitoring available  - Routinely performed  - Used occasionally  - Never performed  Type of concentrations monitored  - Cmin monitoring only  - Cmax monitoring only  - Cmax and Cmin monitoring  Type of patients monitored  all ICU patients  patients with AKI  obese patients  prolonged AG therapy | 53 (96%)  40 (76%)  12 (23%)  1 (2%)  13 (25%)  3 (6%)  36 (69%)  32 (61%)  16 (31%)  10 (19%)  14 (27%) |
| Weight-based AG dosing regimens  TBW IBW ABW | 33 (60%)  12 (22%)  11 (20%) |
| Local AG guidelines | 48 (87%) |
